# Charcot neuroarthropathy in simultaneous kidney–pancreas transplantation: report of two cases

**DOI:** 10.3402/dfa.v4i0.21819

**Published:** 2013-08-29

**Authors:** Jorge Javier del Vecchio, Nicolás Raimondi, Horacio Rivarola, Carlos Autorino

**Affiliations:** 1 Foot and Ankle Section, Department of Orthopaedic and Traumatology, Favaloro Foundation, CABA (Ciudad Autónoma de Buenos Aires), Argentina; 2 Arthroscopy and Sports Traumatology Section, Department of Orthopaedic and Traumatology, Favaloro Foundation, CABA (Ciudad Autónoma de Buenos Aires), Argentina; 3 Prosthetic and Reconstructive Surgery Section, Department of Orthopaedic and Traumatology, Favaloro Foundation, CABA (Ciudad Autónoma de Buenos Aires), Argentina

**Keywords:** arthropathy, neurogenic, Charcot foot, transplant, kidney, pancreas

## Abstract

Charcot neuroarthropathy (CN) is considered a major complication in diabetes mellitus (DM), and it is estimated that 1% of diabetic patients may develop this complication. Simultaneous kidney–pancreas transplantation (SKPT) is one of the most effective therapies for patients with type 1 DM and end-stage diabetic nephropathy. Some cases with a Charcot-modified clinical presentation during the postoperative convalescence period after SKPT have been described. The clinical presentation may condition severe destructive lesions, and good practices include systematic follow-up. Based on the cases described, SKPT is one more entity that might lead to CN ‘foot-at-risk’. The aim of this article is to describe two cases of neuropathic arthropathy with rapid progression in the short term after SKPT.

Charcot neuroarthropathy (CN) is considered a major complication in diabetes mellitus (DM) (1), and it is estimated that 1% of diabetic patients may develop this complication (2). CN typically features two phases: (a) an acute active phase and (b) a stable chronic phase. The acute phase is characterized by erythema and unilateral oedema. A temperature difference of at least 2°C more is detected in the feet (1). The local inflammatory response is considered as the main characteristic expression of the acute phase (3).

Simultaneous kidney–pancreas transplantation (SKPT) is one of the most effective therapies for patients with type 1 (DM) and end-stage diabetic nephropathy (4–6). Some cases with a Charcot-modified clinical presentation during the postoperative convalescence period after SKPT have been described (7), and our goal of this article was to report on two case presentations of neuropathic arthropathy with rapid progression in the short term after SKPT.

## Case report 1

The first patient was a 31-year-old woman with a type 1 DM diagnosed at the age of 7. Patient's medical history consisted of hepatitis C (treated with pegylated interferon) in February 2010, diabetic retinopathy (amaurosis of the right eye; loss of visual acuity in the left eye), recurrent urinary tract infections, amputations from the third to the fifth rays of the right foot, and end-stage renal disease (ESRD) on haemodialysis leading to SKPT in April 2010. Post-transplant relevant clinical analytical data showed glycaemic levels of 106–136 mg% and creatinine levels of 1.4–2.1 mg% while the most routinely used post-transplant immunosuppressants included mycophenolate mofetil (1.4–1.5 g/day), tacrolimus (1–3 mg/day, adjusted according to blood tacrolimus levels), and glucocorticoids such as methylprednisolone for the last 8 months (4–8 mg/day). During the post-transplant follow-up, the patient did not require insulin, had an episode of acute rejection, and was treated with metabolic control, diet, and with a transient correction of immunosuppressants.

**Fig. 1:**
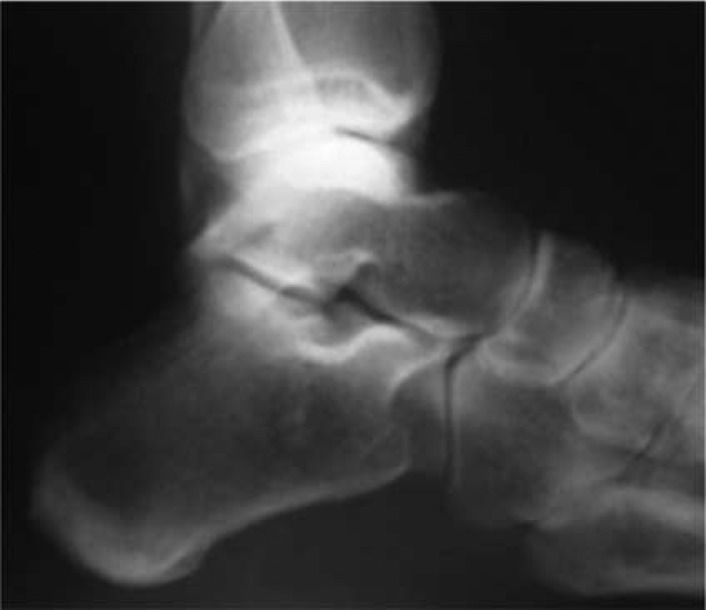
Plain lateral radiographic view without evidence of changes in shape and skeletal structure.

**Fig. 2:**
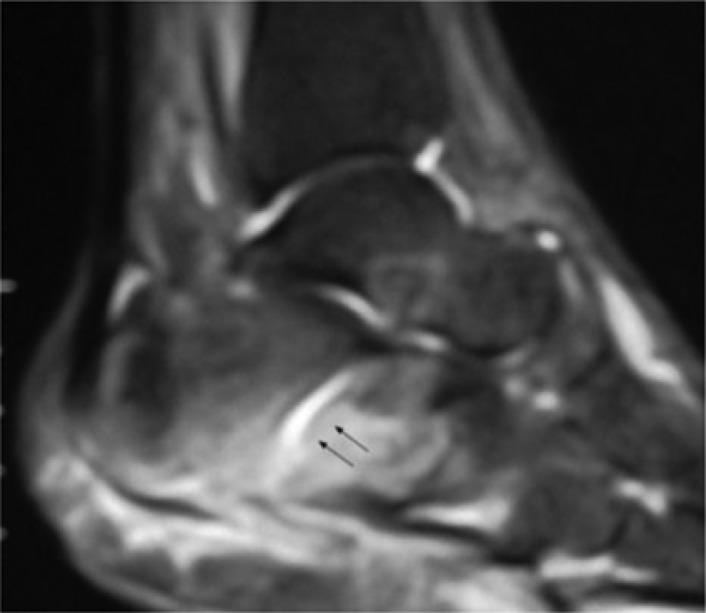
Calcaneal complete medial facet fracture without displacement associated with bone oedema (see indicators).

The patient was being managed with topical negative pressure wound therapy treatment for ulcers in her right heel (posterior, non-weight bearing area) when she had a low-energy trauma in the lateral aspect of her heel due to a fall. At that time, plain radiographs (November 2009) did not reveal any structural bone changes (Fig. 1). One month later, due to persistent pain during the heel contact phase, a magnetic resonance imaging (MRI) was performed, which showed a complete short oblique fracture from the medial aspect of the calcaneus without displacement associated with bone oedema (Fig. 2). The topography of neuroarthropathy involvement was type 5 according to the Sanders and Frykberg classification (8).

**Fig. 3:**
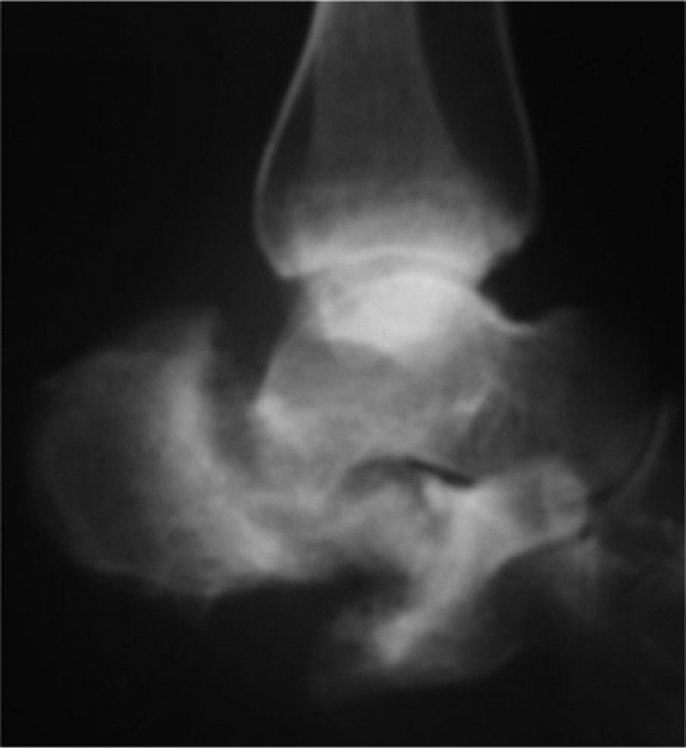
Plain radiographic lateral view showing bone resorption and collapse of the anterior and medial tuberosity of the calcaneus as well as proximal displacement of the greater tuberosity.

**Fig. 4:**
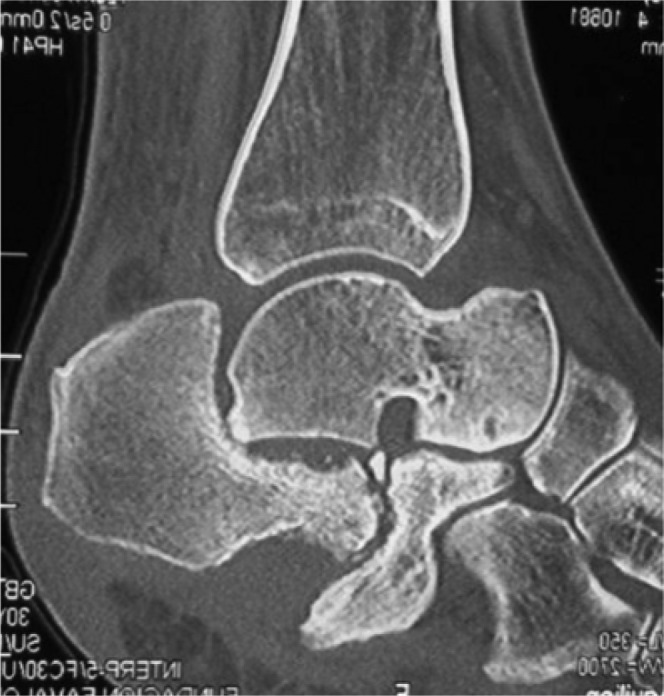
Sagittal view of a computed tomography scan showing pseudoarthrosis and remodelling of the calcaneal tuberosity.

**Fig. 5:**
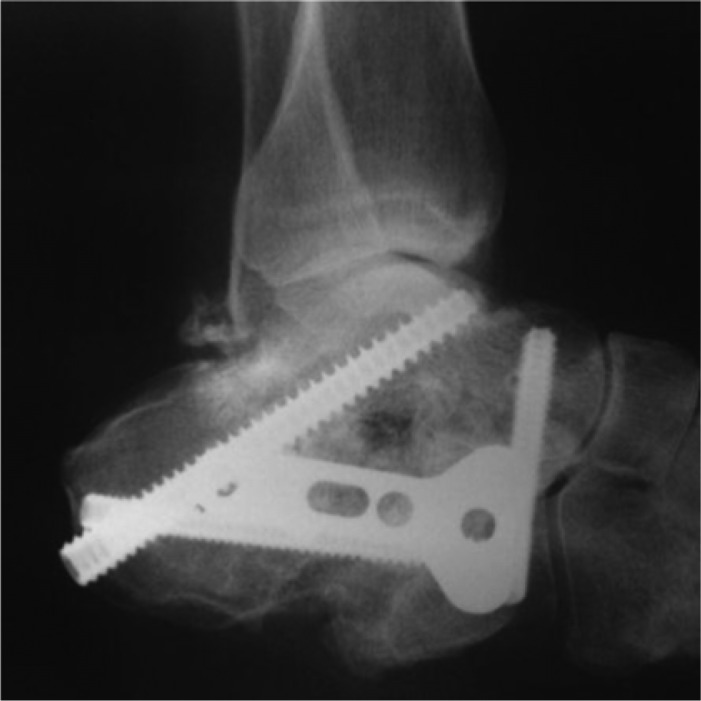
Plain radiographic lateral ankle view showing a subtalar joint arthrodesis with rigid internal fixation consisted of plate and screws.

**Fig. 6:**
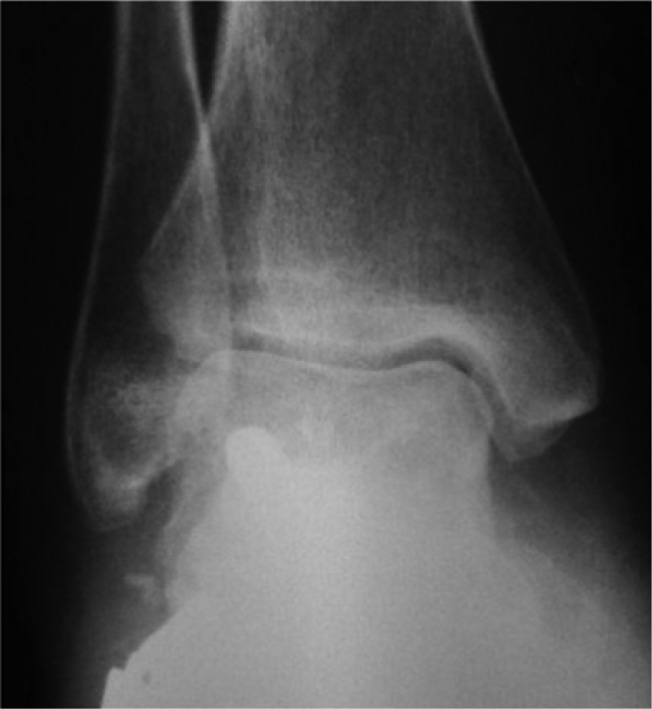
Post-operative antero-posterior ankle view showing a well-aligned ankle and hindfoot.

Non-weight bearing and immobilization with a plaster cast for 8 weeks was then initiated and followed with new plain radiographs and computed tomography scan after the final cast removal. The studies revealed that reabsorption patterns and bone collapse in the anterior and medial tuberosity of the calcaneus as well as proximal displacement of the posterior tuberosity were evidenced due to traction of the Achilles tendon (Figs. 3 and 4). Stabilisation with rigid osteosynthesis using 3.5-mm screws and a T-shaped plate was then performed. Focus consolidation was observed 3 months after the surgery (Figs. 5 and 6).

## Case report 2

The second patient was a 32-year-old woman with a medical history of type 1 DM, sedentarism, hypertension, ESRD without haemodialysis since January 1993, retinopathy and retinal detachment in the right eye, renal and perinephric abscess (2002, left perirenal abscess requiring surgical treatment). Also presents antecedent of SKPT (3 years and 10 months ago) and the donor was an 18-year-old female, who died due to traumatic brain injury after a motor vehicle accident with negative cytomegalovirus serostatus and blood group O+. The patient also underwent a laparoscopic cholecystectomy 6 months after her SKPT for gallbladder lithiasis.

Post-transplant relevant clinical analytical data showed glycaemic levels of 83–85 mg% and creatinine levels of 0.6–0.8 mg%, while the most routinely post-transplant immunosuppressants included mycophenolate mofetil (500 mg–2 g/day), tacrolimus (2–6 mg/day, adjusted according to blood tacrolimus levels), and glucocorticoids such as methylprednisolone for the last 8 months.

In July 2008, the patient consulted our service for pain and deformity in her left foot. On physical examination, a widening of the hindfoot and an apparent length discrepancy due to shortening without any signs of local swelling (neither hyperthermia nor erythema) were seen. The patient also had paraesthesia sensations and preserved sensitivity in the Semmes–Weinstein monofilament testing. The patient stated that she had not had local swelling for the last 6 months but confirmed significant progression of both pain and deformity. Vascular examination using arterial echo Doppler of the lower limbs evidenced the following: mild obstruction (<20%) of the common femoral artery and bilateral superficial artery due to the presence of fibre-lipid plaques. The popliteal, posterior, and anterior tibial arteries depicted smooth walls with normal flow. Bilateral ankle/brachial index was >1 (within normal values).

**Fig. 7:**
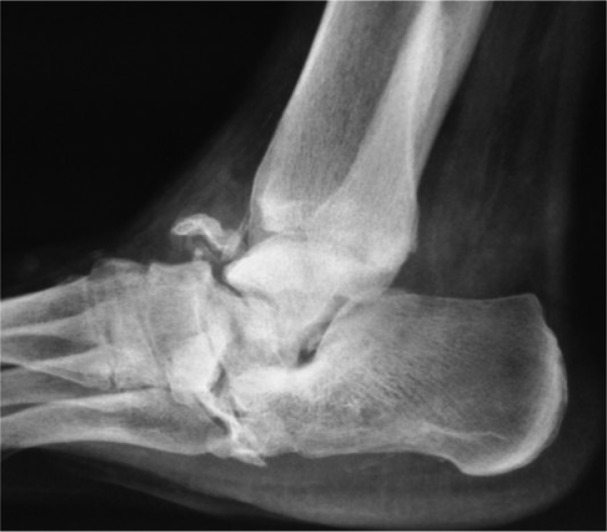
Plain radiographic lateral ankle view showing a collapse of the talar head and neck with resorption of the talus.

**Fig. 8:**
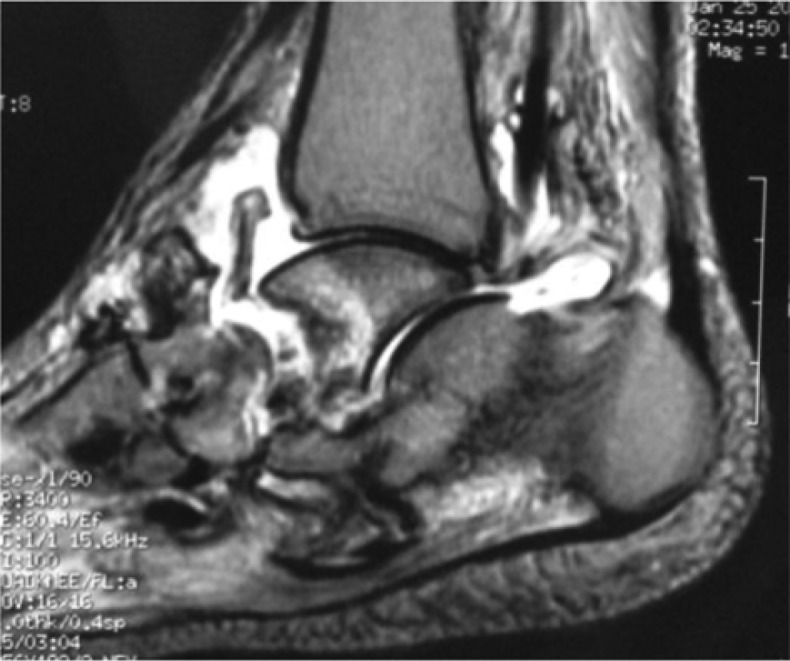
Sagittal view of a magnetic resonance imaging showing the ankle anterior capsule breached, soft tissue oedema and bone oedema in the anterior talar body.

**Fig. 9:**
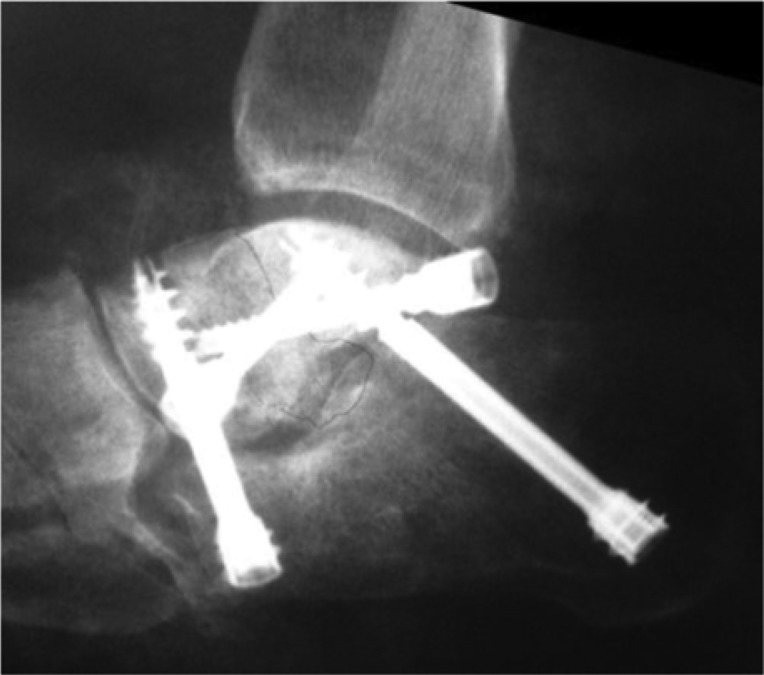
Stabilization by osteosynthesis using 4.5- and 6.5-mm double-threaded screws.

**Fig. 10:**
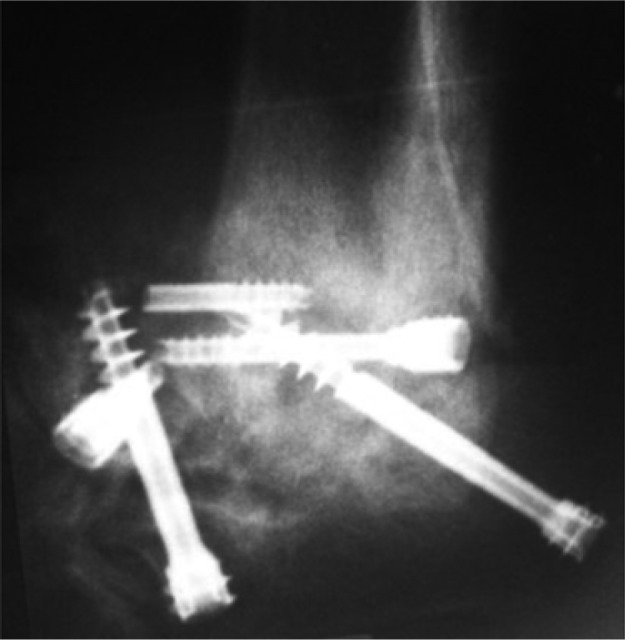
Post-operative plain radiograph showing the mechanical assembly failure and progression.

**Fig. 11:**
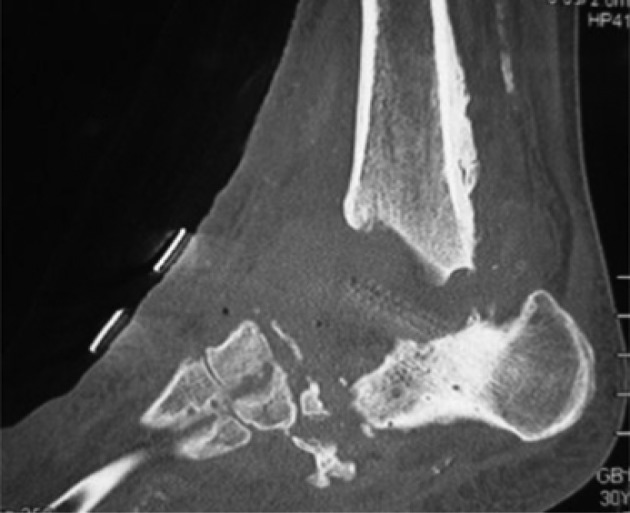
Computed tomography showing absorption (tibial and calcaneal severe bone defects) after removal of osteosynthesis.

**Fig. 12:**
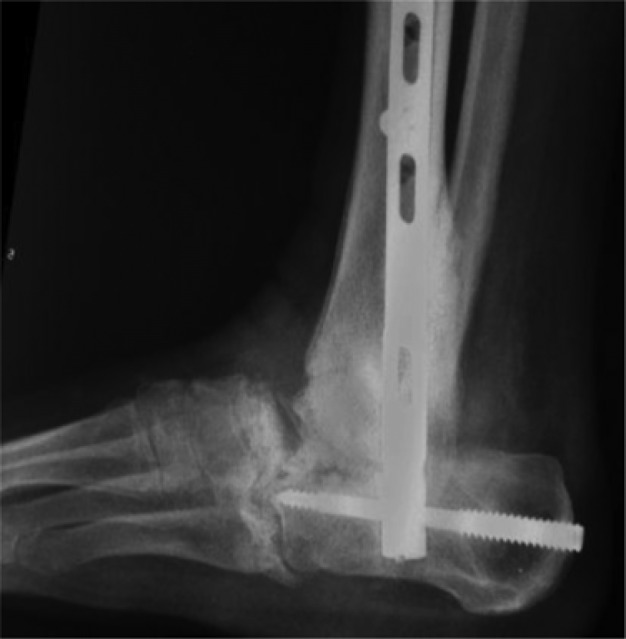
Post-operative outcome of the revisional surgery included the retrograde intramedullary tibio–talo–calcaneal nailing (Panta^®^) for arthrodesis.

The imaging protocol included plain radiographs and MRI that revealed complete reabsorption of the head and neck of the talus, talar body collapse and displacement in plantar flexion (type 4 Sanders and Frykberg classification) (8), and partial reabsorption of the navicular and cuboid (Figs. 7 and 8). Stabilisation was performed by osteosynthesis using 4.5- and 6.5-mm double-threaded screws (Fig. 9). The outcome was unfavourable due to mechanical assembly failure and reabsorption progression (Figs. 10 and 11). Revisional surgery was then carried out with tibio–talo–calcaneal arthrodesis by retrograde intramedullary nailing (Panta^®^) (Fig. 12).

## Discussion

A higher incidence of diabetic foot pathology has been observed in patients with concomitant renal disease, and the outcome is in general suboptimal due to the complications (amputations, mortality) (9). Several specific risk factors in all renal disease categories have been described including ESRD not requiring transplantation (stages 3 and 4) (10); renal disease managed with continuous ambulatory peritoneal dialysis (11); and renal disease managed with kidney transplant and renal disease in patients with type 1 DM managed with SKPT (12, 13).

In the cases studied, only the stable chronic stage of CN (1) was identified clearly. The pathogenesis of such an evolutive pattern is still unknown; at present the hypothesis of the role of immunosuppression still prevails, according to some authors (7). In both cases, destruction was ‘rapid and fulminant, without a leading infectious pattern’ (14), with well-defined disintegration models (15, 16). According to Caldara et al., for patients already affected by severe diabetic complications, tight metabolic control achieved with pancreas transplantation does not prevent the development of CN (17).

The MRI is a significant diagnostic implement when plain radiographs are negative and in the presence of a clinical suspicion of CN. However, MRI demonstrated bone marrow oedema in midfoot and hindfoot areas in 30% of the patients with neuropathic diabetic ulceration and did not predict future CN or osteomyelitis; this type of bone marrow oedema was more common in ESRD (7, 18). According to Valabhji and our brief experience, clinicians should be aware that Charcot can present post-transplantation without the cardinal clinical signs but still lead to deformity (7).

Patients undergoing SKPT are at risk for the development of CN *de novo* as a comorbidity. The dose of glucocorticoids is the main pathogenic factor, and this correlation could be observed in this series (4). According to Rangel et al., foot examination and orthopaedic assessment should be routinely performed even more when risk factors are identified. Immunosuppression regimen based on glucocorticoid minimisation or avoidance should also be considered (4).

The cases described use the ‘foot-at-risk’ concept characterised by baseline comorbidities (DM, severe peripheral vasculopathy, prolonged steroid therapy, etc.). The clinical substrate may condition severe destructive lesions, and good practices include systematic follow-up. Based on the cases described, SKPT is one more entity that might lead to neurogenic arthropathy.
